# Citrullination of fibrinogen: generation of neoepitopes and enhancement of immunostimulatory properties

**DOI:** 10.1186/ar3585

**Published:** 2012-02-09

**Authors:** William H Robinson, Jeremy Sokolove

**Affiliations:** 1 VA Palo Alto Health Care System, Palo Alto, CA 94304 and the Division of Immunology and Rheumatology, Stanford University School of Medicine, Stanford, CA 94305, USA

## 

Rheumatoid arthritis (RA) affects approximately 0.5% of the world population, yet the mechanisms underlying the development and progression of RA remain poorly understood. We are investigating the role of citrullinated fibrinogen as a pathogenic antigen in RA. Using arthritis antigen arrays we demonstrate that citrullinated fibrinogen is one of the earliest targets of the autoantibody response in RA, with autoantibodies against citrullinated fibrinogen appearing up to 10 years prior to the development of clinical arthritis. We further demonstrate that approximately 50% of CCP+ RA patients possess circulating immune complexes containing citrullinated fibrinogen, and that citrullinated fibrinogen containing immune complexes are deposited in human RA synovial tissues. To determine whether citrullinated fibrinogen can induce inflammatory arthritis in mice, we immunized mice with citrullinated fibrinogen and demonstrated that an inflammatory arthritis results and that both T cells and serum can transfer arthritis to naïve mice. Fibrinogen is an endogenous ligand for the innate immune receptor TLR4, and to determine whether citrullination might alter the ability of fibrinogen to bind TLR4 we performed in vitro macrophage stimulation assays with native and citrullinated fibrinogen. We found that citrullinated fibrinogen was ten-fold more potent than native fibrinogen at stimulating macrophage TNF release. Further, macrophage derived from mice deficient for TLR4 or MyD88 did not produce TNF in response to citrullinated fibrinogen. Thus, our results demonstrate a novel mechanism by which anti-citrullinated protein antibodies (ACPA) specifically targeting citrullinated fibrinogen may directly stimulate macrophage TNF production, via co-ligation of TLR4 and Fc-gamma-R. Our findings demonstrate a role for citrullination both in creating neoantigens targeted by the adaptive immune response in RA as well as by increasing the potency of fibrinogen as an endogenous innate immune ligand. These results provide insights into the mechanisms by which anti-citrulline autoimmunity, and specifically the citrullination of fibrinogen, may contribute to both the onset and propagation of inflammation in RA.

